# The therapeutic effects of autologous conditioned serum on knee osteoarthritis: an animal model

**DOI:** 10.1186/s13104-022-06166-1

**Published:** 2022-08-12

**Authors:** Alireza Pishgahi, Majid Zamani, Amir Mehdizadeh, Leila Roshangar, Milad Afkham-Daghdaghan, Behzad Pourabbas, Mehdi Yousefi

**Affiliations:** 1 Stem Cell Research Center, Tabriz University of Medical Sciences, PO Box: 6446-14155, Tabriz, Iran; 2 Department of Medical Laboratory Sciences, Faculty of Allied Medicine, Infectious Diseases Research Center, Gonabad University of Medical Sciences, Gonabad, Iran; 3 Hematology and Oncology Research Center, Tabriz University of Medical Sciences, Tabriz, Iran

**Keywords:** Osteoarthritis, Autologous conditioned serum, Cytokines, Growth factors

## Abstract

**Objective:**

As a progressive chronic condition, osteoarthritis (OA) causes substantial pain and impairment. Secrete proinflammatory cytokines are essential mediators involved in the pathophysiology of OA. In this regard, the clinical effectiveness of autologous conditioned serum (ASC) has been shown through its injection into OA tissues. This study aimed to assess the effectiveness and concentration level of ACS components produced by Nano-carbon glass beads.

Intravenous whole blood was obtained from each New Zealand male rabbit by 10-ml syringes, comprising 33 medical-grade Nano carbon-coated glass beads. Serum retrieving was performed after 6–8 h incubation (37 C, 5% Co_2_), and then centrifuged. The ACS was then injected into OA rabbits to assess its function.

**Results:**

Glass beads-prepared ACS coated with Nano-carbon, induced a huge amount of cytokines and growth factors production. The concentration level of anti-inflammatory cytokines and proinflammatory cytokines was improved throughout Nano-carbon coated glass beads stimulation. ACS also shortened the recovery time and improved the function and mobility of OA rabbits.

We showed that ACS improved the function and mobility of OA rabbits, as well as shortened the recovery time. It is suggested that further studies evaluate this effectiveness.

**Supplementary Information:**

The online version contains supplementary material available at 10.1186/s13104-022-06166-1.

## Introduction

Osteoarthritis (OA) is a common degradative articular cartilage disease, leading to joint dilution, hypertrophic bone modifications, pain, discomfort, and disability [1], thereby putting a significant strain on healthcare systems [2, 3]. According to recent estimates, nearly 67 million Americans over the age of 18 will be diagnosed with OA by 2030, with medical expenses of more than $100 billion [4]. Joint pain is the most frequent manifestation of OA, and the most often affected joints are the hips, hands, back, and knee. The risk of developing OA increases with age, obesity, and past trauma [5, 6]. There are very few pharmacological OA treatment choices, including non-steroidal anti-inflammatory drugs (NSAIDs), analgesics, and the intra-articular injection of hyaluronan or steroids [7, 8]. Unfortunately, no definite therapies for OA are available. The key objectives of modern OA treatment are pain relief and joint function improvements [9].

The pathogenesis and etiology behind this debilitating disease are not very well understood. The interactions between catabolic factors, including interleukine-1 (IL-1), tumor necrosis factor (TNF) alpha, proteinases, and anabolic factors, including insulin-like growth factor (IGF) I and II, affect the complex balance between the development and decomposition of cartilage matrix [6]. In this respect, local cytokine production, which causes hyaline cartilage and its matrix degradation, is one of the principal molecular pathologies [10]. Epidemiological trials demonstrate a direct connection between the development and the existence of inflammatory synovium and tibiofemoral cartilage injury [11, 12]. Thus, secreted proinflammatory cytokines are a serious mediator for increased catabolism of joint tissues implicated in OA. The major proinflammatory cytokines involved in OA pathogenesis are IL-1β, IL-6, and TNF [9]. In this regard, the IL-1 receptor antagonist (IL-1Ra), can theoretically regulate intranet activities of IL-1 and thereby regulate the disease process as a naturally occurring regulator [13, 14].

Endogenous IL-1Ra-enriched autologous conditioned serum (ACS) was designed as a novel injectable substance for OA therapy in the mid-1990s. Meijer et al. [15] reported a rapid elicitation of several anti-inflammatory cytokines, such as IL-1ra, IL-4, and IL-10, as a result of exposing blood to glass beads. Following monocyte activation, the level of IL-13 is also increased, leading to improved IL-1Ra and IL-1β production hindering [16]. Exposing blood to glass beads also facilitates the extraction of many growth factors, including transforming growth factor-beta (TGF-β), Platelet-derived growth factor (PDGF), and IGF-1, in the liquid phase of blood [17]. The current study aimed to evaluate the macroscopic and microscopic effect of glass beads-produced ACS coated with Nano-carbon substances in OA rabbits. Moreover, the concentration level of cytokines and growth factors produced by polished glass beads and Nano-carbon-coated glass beads was also compared.

## Main text

### Materials and methods

#### Animals

The New Zealand Male rabbits (2.2 ± 0.2 kg) were purchased from Pasture Institute, Tehran, Iran, and held at 22 ± 1 °C in the Stem Cell Research Center, Tabriz University of Medical Sciences. In a metabolic cage under a light/dark 12/12 h cycle, every animal had free access to food and water. The experiments were conducted in compliance with national standards and endorsed by the local ethics committee. Collagenase was used to develop OA in the knee of rabbits. The OA animals were divided into two ACS-treated and normal saline-treated groups.

#### ACS preparation

From each rabbit, 10 ml of whole blood was taken (without anti-coagulants) by syringes containing 33 medical-grade Nano carbon-coated glass beads with a diameter of 2.5 mm and a surface area of 21 mm^2^. In an aseptic condition, whole blood was incubated at 37 °C, and 5% CO_2_ for 6–8 h. Then, the serum was recovered and centrifuged at 1000 g for 10 min. The prepared ACS was stored at −20 °C until use. The control serum was immediately centrifuged without any incubation, and preserved at −20 °C until use.

#### ACS safety tests on serum

External, certified clinical laboratories evaluated the existence of microbial pollutants (bacteria, fungi, and mycoplasma) and serologic criteria in serum generated by syringes.

#### ACS ELISA tests

The ACS cytokines and growth factor levels were evaluated by ELISA in a pre-pilot experiment. The blood samples of 10 different rabbits were used, and ELISA tests were performed according to the manufacturer’s instruction for the analysis of basic fibroblast growth factor (b-FGF), epidermal growth factor (EGF), PDGF-AB, IGF-1, TGF-β, IL-1Ra, IL-1β, IL-4, IL-6, IL-8, IL-10, and IL-13 (MyBioSource, San Diego, California).

#### Macroscopic evaluation of ACS effects

Rabbits were put in a 2 square meter area to assess the improvement of rabbit lameness in the knee. Two physiotherapists who were oblivious of animal health assessed the flexion, gait, extension, and rotation of animals for 28 days for 30 min. The Knee Position Comparison Scale was used for the comparison of a healthily extended and flexible knee. The damaged knee had abnormal, non-weight bearing extension and flexibility. When the rabbit’s knee extension and flexibility returned to the normal day, it was taken into account as the knee recovery time. The degeneration status of cartilage was macroscopically assessed. For the assessment of gross morphological changes in the knee joint, rabbits were anesthetized, and the knee joint was removed.

#### Microscopic and histological evaluation of ACS effects

Hematoxylin and eosin (H&E) staining were used for the microscopic analysis of rabbit knee cartilage destruction. For this purpose, 10% of neutral buffered formalin was used for the fixation of separate bone samples. The samples were then decalcified using 20% ethylenediaminetetraacetic acid (EDTA) and soaked in paraffin. Then, 5 μm sections were prepared by microtone and stained by H&E. Samples were assessed by a pathologist without any information about the intervention group and controls. At the end of the study to avoid further harassment to animals we began to euthanized them. To do this, the animals were injected with the Pentobarbital overdose  ≥ 150 mg/kg intravenously.

#### Statistical analysis

SPSS Version 15.0 (SPSS Inc., Chicago, IL, USA) was applied to perform data analysis. The mean level of studied parameters between ACS-treated (polished glass beads and coated glass beads) and baseline were compared by paired t-test. P < 0.05 was considered statistically significant.

### Results

#### Nano-carbon glass beads improved the cytokines and growth factors production in ACS

**Fig. 1:**
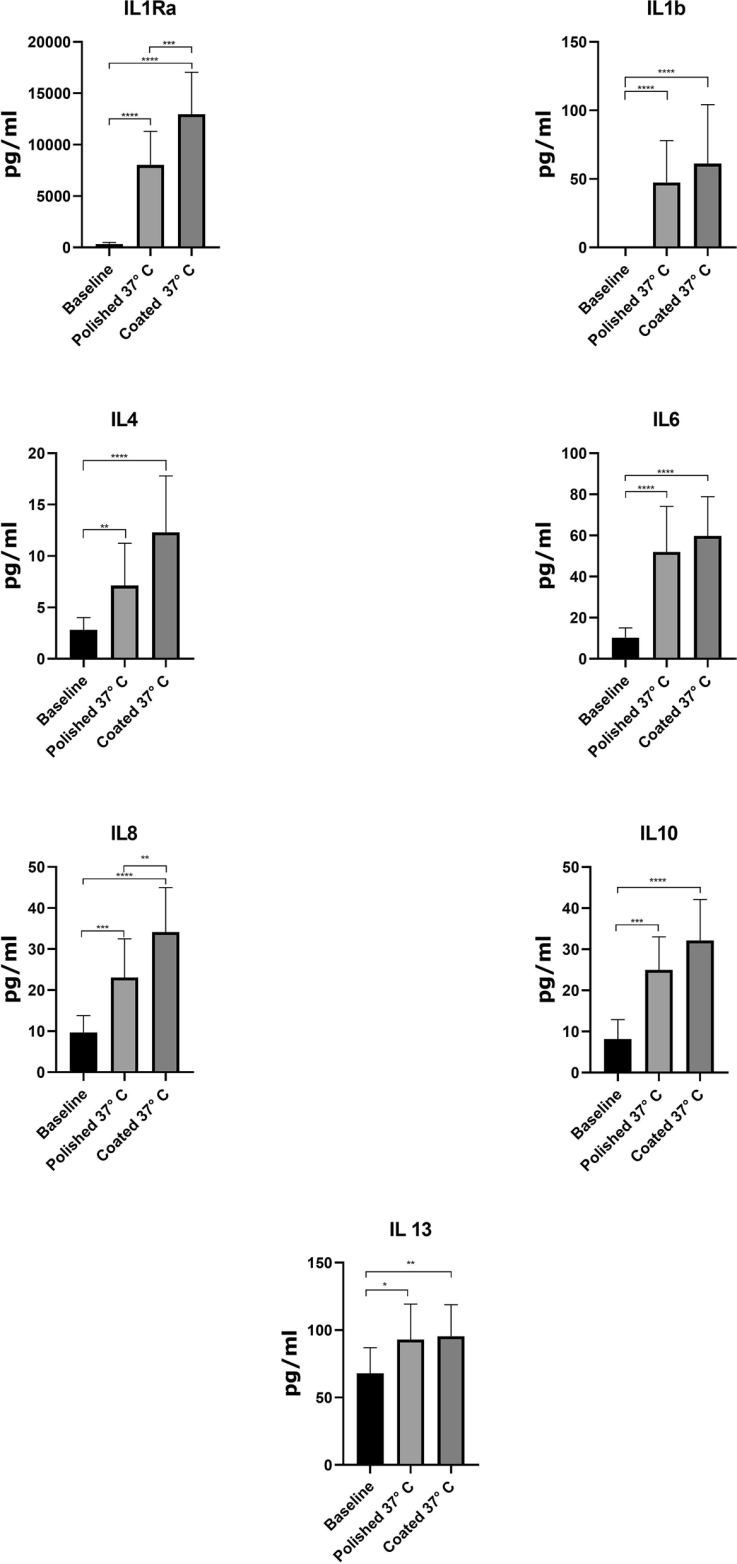
Cytokines level in Nano-carbon-stimulated or polished glass beads ACS. Data are presented as mean ± standard division (SD). P < 0.05 was considered statistically significant

**Fig. 2:**
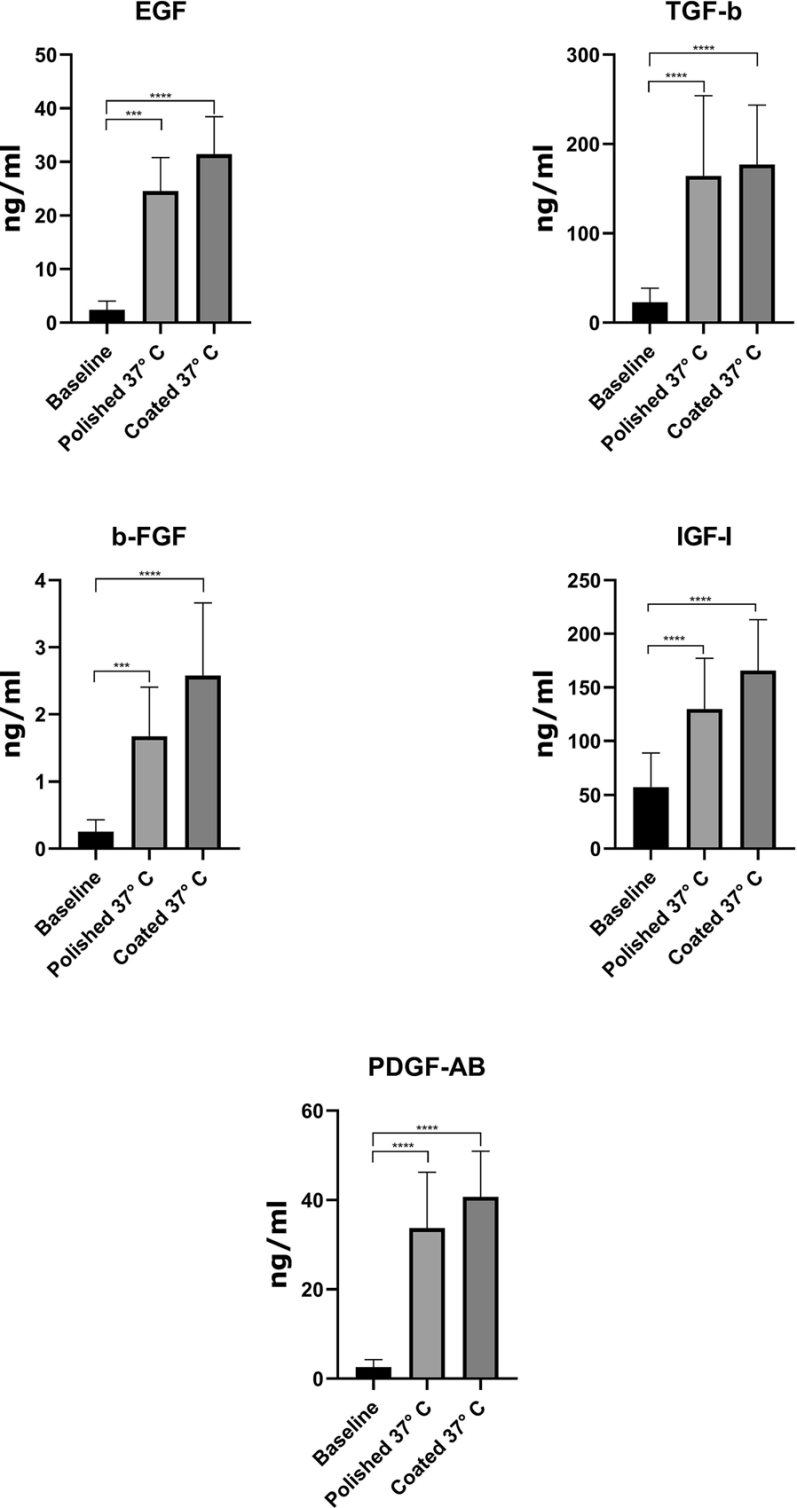
Growth factors concentration level in Nano-carbon-stimulated or polished glass beads ACS. Data are presented as mean ± standard division (SD). P < 0.05 was considered statistically significant

The results of the ELISA test revealed that the concentration of cytokines, either anti-inflammatory (IL-1Ra, IL-4, IL-10, and IL-13) or proinflammatory (IL-1β, IL-6, and IL-8) were improved in ACS produced coated glass beads as compared to polished glass beads (Fig. 1 and Additional file 1). The concentration level of anti-inflammatory cytokines (IL-1Ra, IL-4, IL-10, and IL-13) stimulated by the polished glass were 8028 ± 3269, 7.11 ± 4.12, 24.99 ± 8.05, and 92.92 ± 26.36 while the concentration level of these cytokines stimulated by Nano-carbon-coated glass beads were 12953 ± 4090, 12.3 ± 5.47, 32.14, 32.14 ± 9.948, and 95.47 ± 23.33, respectively. Although polished glass beads provoked proinflammatory cytokines (IL-1β, IL-6, and IL-8), the concentration levels of proinflammatory cytokines induced by Nano-carbon-coated glass beads were relatively higher with the number of 61.19 ± 43.06, 59.76 ± 19.14, and 34.16 ± 10.80, respectively. Additionally, the concentration of growth factors including b-FGF, EGF, PDGF-AB, IGF-1, and TGF-β were significantly increased in ACS exposed to Nano-carbon coated glass beads, as compared to polished glass beads alone. The concentration of growth factors for Nano-carbon coated glass beads were 2.577 ± 1.085, 31.44 ± 7.007, 40.71 ± 10.20, 164.5 ± 47.09, and 177.1 ± 66.54 while the concentration for polished glass beads was 1.673 ± 0.73, 24.57 ± 6.24, 33.74 ± 12.49, 129.8 ± 47.41, and 164.3 ± 89.84, respectively (Fig. 2 and Additional file 1).

#### ACS improved the macroscopic and lameness condition of the cartilage

**Fig. 3:**
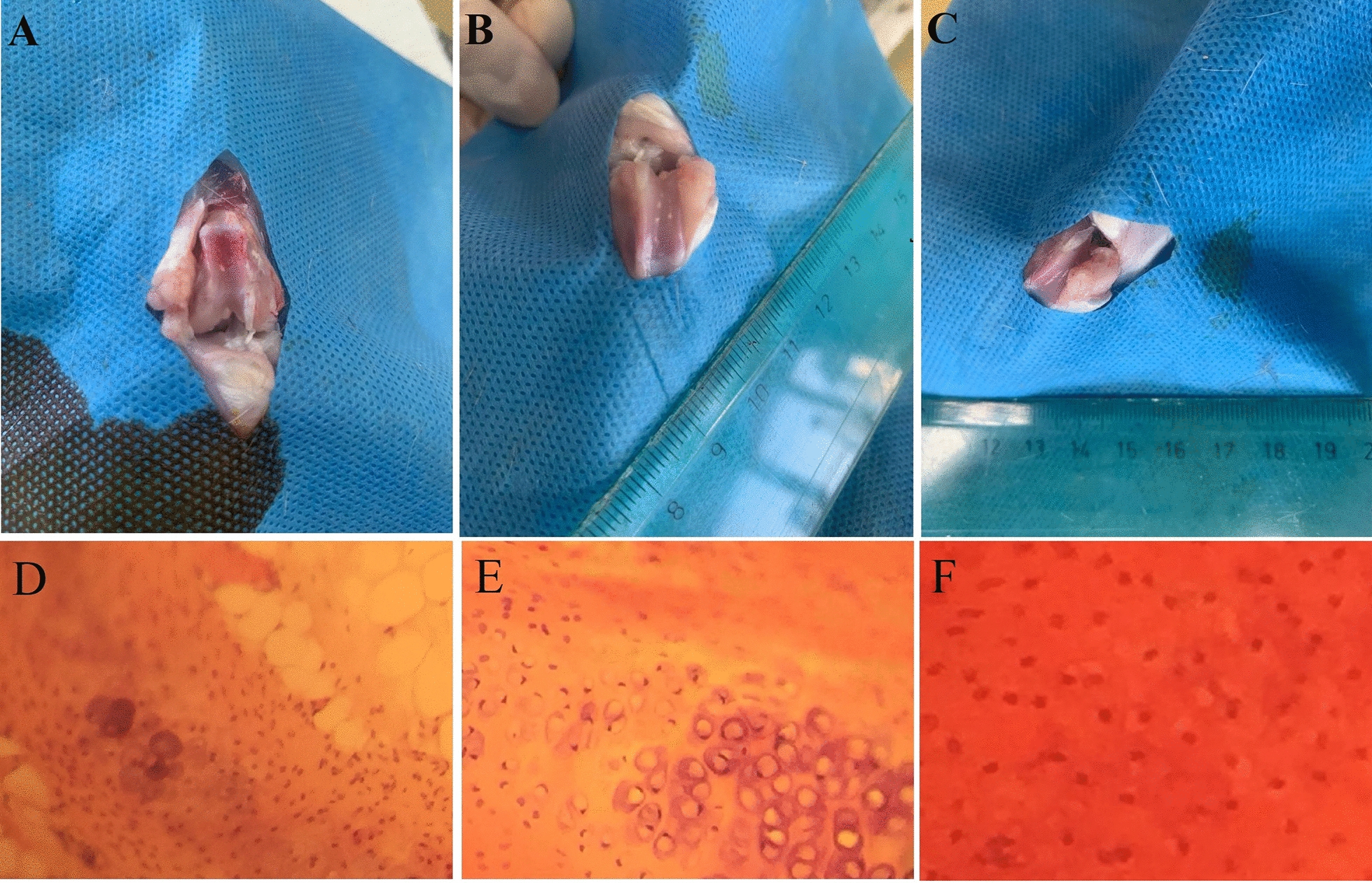
Gross morphological and histological sample stained with hematoxylin and eosin. **A** Day 1 experiment, **B** Normal saline group at day 28, **C** Autologous conditioned serum group at day 28, **D** Day1 experiment, **E** Normal saline group at day 28, and **F** autologous conditioned serum group at day 28

In the ACS-treated group, on the 14th ± 2 day, and the normal saline-treated group on the 20th ± 4 day, non-weight bearing of the injured knee was relieved and the knee flexibility and extension returned to normal, which demonstrated a considerable difference between the two groups. Macroscopic evaluation of the knee joint revealed that in the ACS group, the severity of knee joint destruction was less than in the control group. Less surface irregularity was found with cartilage in the ACS treatment group. Also, more cell growth and greater regeneration were observed in this group, in comparison to the normal saline treatment group (Fig. 3 A–C).

#### ACS improved the microscopic and histological condition of cartilage degeneration

Outcomes evaluation of overall cartilage degeneration score revealed a lower score for the ACS-treated group compared to the normal saline-treated group. Additionally, the ACS-treated group showed normal cellularity in transitional and radial zones, as well as better chondrocyte cloning in the former zone, compared to the normal saline-treated group (Fig. 3 D–F).

### Discussion

A variety of functional and biological disabilities are associated with osteoarthritis. Synoviocyte-excreted cytokines and intraarticular environment growth factors, as well as activated immune cells, partly mediate OA associated cartilage deterioration [18, 19]. This leads to the generation of both destructive and pro-inflammatory intracellular factors which encourage further degradation of hyaline cartilage [20]. Pro-inflammatory cytokine IL-1β has a central function in OA progression through triggering inflammatory and catabolic cascade processes. Deterioration of the articular cartilage matrix is controlled by IL-1β and TNF. Along with IL-1β and TNF, other factors such as LIF, IL-21, IL-18, IL-17, IL-15, IL-8, and chemokines have also been involved in OA pathogenesis [9, 21]. IL-1ra is a competing IL-1 antagonist with a Type I and II IL-1 relation [22].

In the early 1980s, it was suggested that IL1 inhibitors can be used therapeutically in certain diseases [23, 24]. The strategies for prohibiting IL-1 biological function include using soluble forms of IL-1 receptors, IL-1Ra, and cytokines IL-4, IL-10, and IL-13 [9]. Injecting ACS into affected tissues has also demonstrated therapeutic efficacy for the prevention of osteoarthritis, disk prolapse, lumbar stenosis, and muscle injuries in animal models and human clinical trial studies [17]. This treatment in many European countries is currently available for patients and is still more commonly applied for equine OA [25]. Baltzer et al. [26] have shown that ACS is effective and preventive against painful knee OA. In parallel to previous studies, our study also demonstrated that Nano-carbon glass beads-stimulated ACS was capable to improve flexibility and extension of OA rabbits, as well as total cartilage degeneration reduction and shortening the recovery time.

The secretion of ACS repeatedly increases the volume of growth factors (TGF-β, IGF-1, bFGF, and PDGF-AB) and anti-inflammatory cytokines (IL-10, IL-1Ra, and IL-1β) [17]. Another study in 2010 revealed that increased amounts of anti-inflammatory cytokines (IL-1Ra and IL-10) and pro-inflammatory cytokines (IL-1β, IL-6, and TNF-α) were achieved after ACS injection; However, serum conditioning seems to exert no net effect on cartilage metabolism [21]. It has been revealed that blood-derived ACS is rich in IL-1Ra, IL-4, IL-10, and IL-13 [15]. Additionally, ACS can also improve and hinder IL-1Ra and IL-1β production, respectively [15, 16]. Our results demonstrated that blood exposure to Nano-carbon-coated glass beads provokes a vigorous, prompt improvement in the synthesis of many anti-inflammatory (IL-1Ra, IL-4, IL-10, and IL-13), and proinflammatory (IL-1β, IL-6, and IL-8) cytokines, and growth factors (EGF, bFGF, IGF-1, PDGF-ab, and TGF-β).

Adequate levels of IL-1Ra are necessary for intra-articular availability. Since IL-1β is known to be functional at low levels, relatively huge amounts of IL-1Ra are needed to inhibit the effect of IL-1β [27]. In vivo studies have demonstrated the increased level of IL-1Ra in an equine synovial fluid almost 35 days after the last ACS injections [25]. It was determined that the IL-1/IL1Ra ratio of the joint was 1:170. For IL-1 inhibition, the minimum required IL-1/IL-1Ra ratio is 1:10 [19]. Thus, ACS injection can strongly inhibit the bioactivities of IL-1 [22]. Moreover, our findings revealed a higher concentration level of IL-1Ra in Nano-carbon glass beads-prepared ACS, compared to polished glass beads. Besides, the concentration levels of all cytokines and growth factors were considerably higher in ACS stimulated by coated glass beads, compared to glass beads alone.

### Conclusion

Prepared Nano-carbon-coated ACS comprises a huge amount of growth factors and cytokines, indicating the higher stimulatory effects on cells. We also showed that ACS improved the function and mobility of OA rabbits, as well as shortened the recovery time. It is suggested that future studies be directed on conducting randomized controlled trials capable to confirm the results of pilot clinical studies and investigating ACS mechanisms of action.

### Limitation

Although the ACS manufacturing process has been found to consistently raise IL-1Ra and other variables, the methods by which the effects are mediated are not entirely known. The observed clinical impact may be explained by a large number of synergistic, active therapeutic molecules, but its long-term durability is more difficult to explain.

## Supplementary Information

**Additional file 1: Table S1.** Concentration of cytokine and growth factors in Autologous conditioned serum.

## Data Availability

Raw data were generated at Tabriz University of Medical Sciences. Derived data supporting the findings of this study are available from the corresponding author on request.

## Abbreviations

ACS: Autologous conditioned serum
b-FGF: Basic fibroblast growth factor
ELISA: Enzyme-linked immunosorbent assay
EGF: Epidermal growth factor
EDTA: Ethylenediaminetetraacetic acid
H&E: Hematoxylin and eosin
IL1-Ra: IL-1 receptor antagonist
IGF: Insulin-like growth factor
IL: Interleukin
LIF: Leukemia inhibitory factor
NSAIDs: Non-steroidal anti-inflammatory drugs
OA: Osteoarthritis
PDGF: Platelet-derived growth factor
TGF-β: Transforming growth factor-beta
TNF: Tumor necrosis factor
