# Supplementary material for: The therapeutic effects of autologous conditioned serum on knee osteoarthritis: an animal model
Source: BMC Res Notes. 2022 Aug 12;15:277. doi: 10.1186/s13104-022-06166-1 (PMC9373483; doi:10.1186/s13104-022-06166-1)
Supplement: Supplementary file 1 — Additional file 1: Table S1. Concentration of cytokine and growth factors in Autologous conditioned serum. [file 13104_2022_6166_MOESM1_ESM.docx]

Table S1. Concentration of cytokine and growth factors in Autologous conditioned serum.

| cytokine | Baseline (pg/ml) | Polished (pg/ml) | Coated (pg/ml) |
| --- | --- | --- | --- |
| IL1Ra | 345/9 ± 146/3 | 8028 ± 3269 | 12953 ± 4090 |
| IL1b | <3.9 ± <3.9 | 47/38 ± 30/52 | 61/19 ± 43/06 |
| IL4 | 2/83 ± 1/17 | 7/11 ± 4/12 | 12/3 ± 5/47 |
| IL6 | 10/28 ± 4/786 | 51/95 ± 22/19 | 59/76 ± 19/14 |
| IL8 | 9/690 ± 4/122 | 23/05 ± 9/447 | 34/16 ± 10/80 |
| IL10 | 8/14 ± 4/77 | 24/99 ± 8/05 | 32/14 ± 9/948 |
| IL13 | 67/99 ± 18/89 | 92/92 ± 26/36 | 95/47 ± 23/33 |
| Growth factor | Baseline (ng/ml) | Polished (ng/ml) | Coated (ng/ml) |
| EGF | 2/38 ± 1/63 | 24/57 ± 6/24 | 31/44 ± 7/007 |
| TGF-b | 22/88 ± 15/69 | 164/3 ± 89/84 | 177/1 ± 66/54 |
| b-FGF | 0/254 ± 0/176 | 1/673 ± 0/730 | 2/577 ± 1/085 |
| IGF | 54/82 ± 25/28 | 129/8 ± 47/41 | 164/5 ± 47/09 |
| PDGFAB | 2/57 ± 1/71 | 33/74 ± 12/49 | 40/71 ± 10/20 |
